# Developmental validation of the novel six-dye Goldeneye^TM^ DNA ID System 35InDel kit for forensic application

**DOI:** 10.1080/20961790.2021.1945723

**Published:** 2021-08-28

**Authors:** Qi Yang, Huan Yu, Yiling Qu, Xiaochun Zhang, Ruocheng Xia, Ziwei Wang, Rui Tan, Lei Xiong, Shihan Xi, Jun Wu, Yuzhen Gao, Suhua Zhang, Chengtao Li

**Affiliations:** a Shanghai Key Laboratory of Forensic Medicine, Shanghai Forensic Service Platform, Academy of Forensic Sciences, Ministry of Justice, Shanghai, China; b Department of Forensic Science, Medical School of Soochow University, Suzhou, China; c Department of Forensic Medicine, School of Basic Medical Science, Wenzhou Medical University, Wenzhou, China; d School of Basic Medicine, Inner Mongolia Medical University, Hohhot, China; e Clinical Medical School, Inner Mongolia University for the Nationalities, Tongliao, China; f PEOPLESPOTINC, Beijing, China

**Keywords:** Forensic sciences, forensic genetics, insertion and deletion polymorphism (InDel), 35InDel kit, developmental validation, capillary electrophoresis (CE)

## Abstract

Insertion/deletion polymorphisms (InDels) have been treated as a prospective and helpful genetic marker in the fields of forensic human identification, anthropology and population genetics for the past few years. In this study, we developed a six-dye multiplex typing system consisting of 34 autosomal InDels and Amelogenin for forensic application. The contained InDels were specifically selected for Chinese population with the MAF ≥ 0.25 in East Asia, which do not overlap with the markers of Investigator^®^ DIPplex kit. The typing system was named as Goldeneye^TM^ DNA ID System 35InDel Kit, and a series of developmental validation studies including repeatability/reproducibility, concordance, accuracy, sensitivity, stability, species specificity and population genetics were conducted on this kit. We confirmed that the 35InDel kit is precise, sensitive, species specific and robust for forensic practice. Moreover, the 35InDel kit is capable of typing DNA extracted from forensic routine case-type samples as well as degraded samples and mixture samples. All markers are proved to be highly polymorphic with an average observed heterozygosity (He) of 0.4582. The combined power of discrimination (CPD) is 0.999 999 999 999 978 and the combined power of exclusion in duos (CPE_D_) and trios (CPE_T_) are 0.978 837 and 0.999573, respectively, which are higher than those of the Investigator^®^ DIPplex kit. Thus, the Goldeneye^TM^ DNA ID System 35InDel kit is suitable for forensic human identification and could serve as a supplementary typing system for paternity testing.

Supplemental data for this article is available online at https://doi.org/10.1080/20961790.2021.1945723 .

## Introduction

Short tandem repeat, also known as STR, is the golden standard for human forensic identification and paternity testing [1–5]. Nevertheless, relatively long amplicon sizes, high mutation rate and genotyping artifacts such as stutter peaks render limitations in genotyping DNA samples with an inferior quality or finite quantity [2,6–9]. Single-nucleotide polymorphisms (SNPs) and insertion/deletion polymorphisms (InDels), the biallelic makers, are also suitable for forensic identification and kinship ana­lysis since they possess several merits including ubi­quity throughout the genome, low mutation rates, high interpopulation variability and short analyzed amplicons that can be adopted for degraded DNA samples [2,10–12]. SNP assays usually require complex and expertise genotyping methods (i.e. SNaPshot technology [13], pyrosequencing [14] and massively parallel sequencing [15], etc.), while the analysis of InDel only demands a routine PCR-to-CE strategy which is compatible with STR operating platform and endows InDels with vaster application prospects in forensics. On account of the aforementioned features of InDels, they can serve as useful supplementary or stand-alone assays for human identification [2,13].

Ever since InDels have been mapped into human genetic variation, an increasing number of multiplex amplification systems using Indels have been constructed for different forensic implementation. Research focus on InDels including personal identification [16,17], paternity testing [18,19], inference of biogeographic ancestry [20,21], disease association study in cardiac sudden death [22,23], genotyping of human tumor tissues [24] and skeletal remains [25]. Hitherto, the Investigator^®^ DIPplex kit (Qiagen, Germany) is the first and the only commercial forensic InDel kit used for simultaneous amplification of 30 autosomal biallelic InDel [26]. Numerous validation studies have been conducted on the Investigator^®^ DIPplex kit to assess the InDel polymorphisms in different populations worldwide [16]. Statistical results confirmed that the InDel markers in the Investigator^®^ DIPplex kit were highly polymorphic in European populations [16,27], while relatively low polymorphism was observed in most of these loci in the Chinese Han population as well as other Chinese ethnic minorities [28–31]. Hence, the Investigator^®^ DIPplex kit is not recommended for paternity or kinship investigations in Chinese population but can be applied as a complementary tool for autosomal STR testing [32]. Besides, consistent and reproducible imbalance between heterozygote peaks and stably shifted mobility variants were observed in certain markers of the Investigator^®^ DIPplex kit, which may decrease the accuracy of the InDel interpretation [26,33].

To maximize the human identification effectiveness of InDel in Chinese population, novel InDels with higher heterozygosity levels should be identified to improve the marker set. In the present study, we select 34 autosomal InDel loci with the minimum allele frequency (MAF) ≥ 0.25 in East Asia, which show no overlap with the InDel markers from the Investigator^®^ DIPplex kit [34,35]. Based on the six-dye fluorescence labeling technology, a multiplex InDel kit containing all the above InDels is constructed and commercialized for Chinese population [36]. This typing system is denominated as Goldeneye^TM^ DNA ID System 35InDel kit (35InDel kit).

Developmental validation studies were performed to demonstrate the effectiveness of the 35InDel kit. Following the “Validation Guidelines for Forensic DNA Analysis Methods” proposed by the Scientific Working Group on DNA Analysis Methods (SWGDAM) [37], the studies for the repeatability/reproducibility, accuracy, sensitivity, stability, species specificity, mixture and population genetics analysis were conducted. Besides, general performance tests on routine case-type samples and degraded samples were also carried out to assess the feasibility of forensic implementation. In a nutshell, the reliability and robustness of the 35InDel kit are manifested in the validation studies.

## Materials and methods

### Marker selection and primer design

The InDel markers of this kit were selected based on the previously established InDel systems in our laboratory [38] and the 1000 Genomes Project (phase 3) (https://www.ncbi.nlm.nih.gov/variation/tools/1000genomes/) [39]. All candidate markers were screened based on the following prediction criteria: (i) human autosomal bi-allelic InDels positioned in the intron areas, (ii) the MAF in East Asia greater than 0.25, (iii) allelic length variation between 3 and 30 bp, (iv) InDel markers in the same chromosome were unlinked (physical distance between InDel markers positioned on a same chromosome no less than 10 cM), and (v) all selected InDel markers could be amplified in a multiplex reaction. Detailed information of the markers is listed in Supplementary Table S1. PCR primer of each InDel marker was designed using Primer premier v5.0 (San Francisco, CA, USA) and Oligo v6.0 (Colorado Springs, CO, USA), applying the following criteria: (i) primer length of 15–30 bp, (ii) PCR amplicon length range between 70 and 250 bp, (iii) Tm value range between 55 °C and 65 °C, and (iv) optimum GC content of 40%–60%. Self-complementarity, false priming, cross dimmer, hairpin secondary structures and inter-primer compatibility among the designed primer were analyzed by AutoDimer software. Non-specific hybridization in other genome regions was also checked by the National Center for Biotechnology Information (NCBI) Basic Local Alignment Search Tool (BLAST) at http://blast.ncbi.nlm.nih.gov/Blast.cgi.

**Table 1.** Combined power of discrimination (CPD) of different multiplex InDel system in Chinese Han population.

Multiplex	Number of markers	Sample origin	Sample size	CPD	Reference
Qiagen Investigator^®^ DIPplex	30	Beijing	210	0.999 999 999 985	[56]
	30	Eastern China	565	0.999 999 999 982	[57]
	30	Shanghai	565	0.999 999 999 982	[32]
	30	Chengdu	129	0.999 999 999 987 8	[29]
	30	Jiangsu	501	0.999 999 999 98	[31]
	30	Hainan	238	0.999 999 999 964 6	[34]
SifaInDel 45plex system	27	Changzhou	502	0.999 999	[38]
35InDel kit	35	Southern China	262	0.999 999 999 999 999	Present study
AGCU InDel 50 kit	50	Guangdong	203	0.999 999 999 999 999	[58]

**Figure 1.**
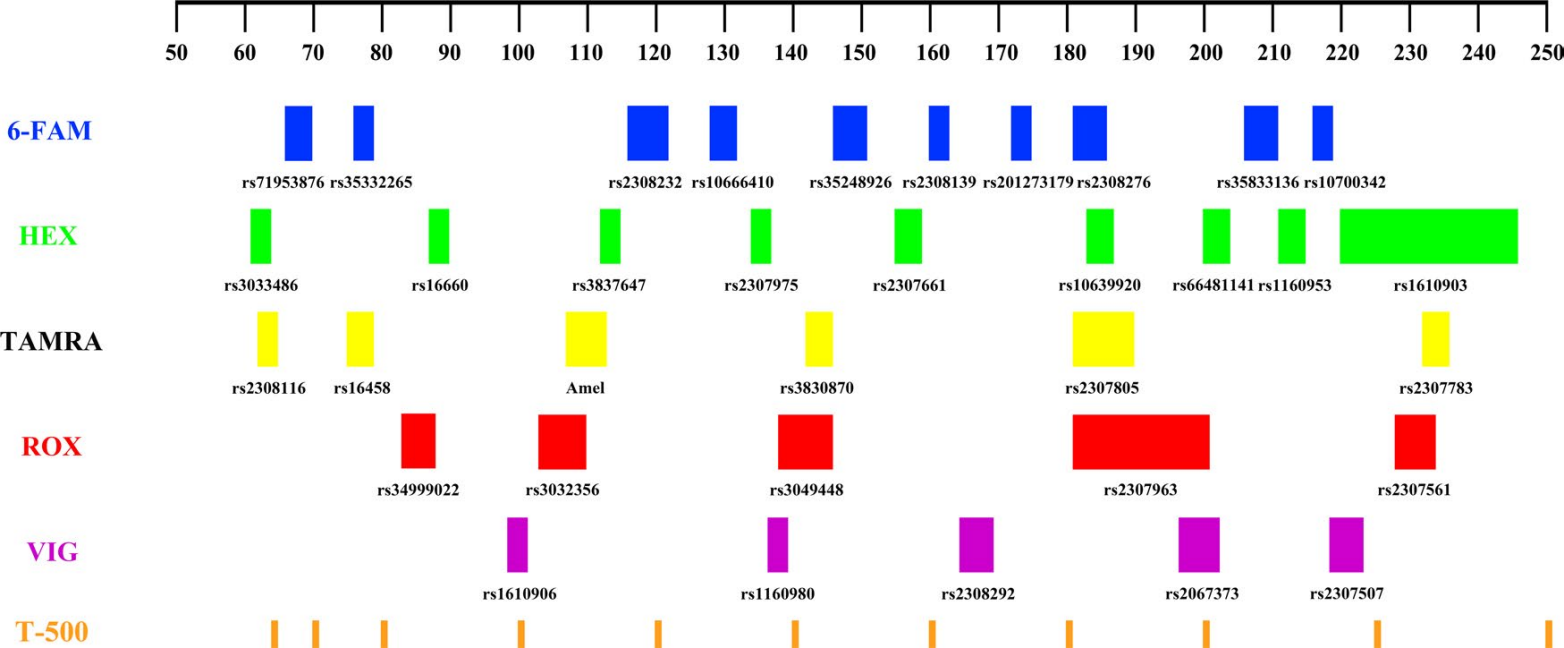
The fluorescence allocation schematic diagram of 35InDel kit.

The primer pair for each InDel maker was initially amplified in a single-plex PCR reaction to evaluate the genotyping performance. PCR amplification was performed in a 12.5 μL final reaction volume, including 1 μL of DNA template (2 ng/μL), 7.5 μL of 2× PCR Master Mix (Qiagen, Hilden, Germany), 1.5 μL of 5 × Q-Solution (Qiagen), 1 μL of 0.2 μmol/μL PCR primers, and 4 μL of Deionized water. The thermal cycling conditions consisted of an initial step at 95 °C for 15 min; 30 cycles of 94 °C for 30 s, 58 °C for 90 s, and 72 °C for 90 s; and a final extension at 60 °C for 60 min. Subsequently, primers for each locus have been optimized and redesigned to accommodate multiple amplification. The concentration of each primer in the final multiplex system was optimized according to the genotyping profiles. Eventually, all selected markers were arranged into five groups based on the allele size and amplicon length, and five fluorescent dyes were labeled at the forward primer of each locus: 6-FAM (blue), HEX (green), TAMRA (yellow), ROX (red) and VIG (purple). T-500 (orange) was used to label the internal size standard. The fragments of the internal lane standard T-500 are 65, 70, 80, 100, 120, 140, 160, 180, 200, 225, 250, 275, 300, 330, 360, 390, 420, 450, 490 and 500 bp, respectively. A schematic diagram of the fluorescence allocation is shown in Figure 1.

### Allele ladder and size precision

Population samples in this study were amplified by non-fluorescent labeled primers. The amplification products of different genotypes at each InDel locus were purified and cloned in plasmid. The recombinant plasmids were amplified and mixed with allelic PCR products of each locus to produce a single allelic ladder. For the formation of allelic ladders of the 35 InDel loci, the concentration of each allelic ladder was balanced according to the peak height ratio. Size precision was assessed by running 24 injections of an allelic ladder and calculating the standard deviation in the fragment size of each allele on a 3500 Genetic Analyzer (Thermo Fisher Scientific, Carlsbad, CA, USA). Panel and Bin files were then compiled based on the above data with the allele range of ±0.5 bp.

### Multiplex PCR amplification and capillary electrophoresis (CE)

After optimization of reaction components, the single-tube multiplex PCR reaction was performed in a volume of 10 μL, containing 2.5 μL of 5 × PCR Reaction Master Mix IV (Goldeneye Co. Ltd), 2 μL of optimal primer mix and 1 ng genomic DNA. PCR amplification was conducted on GeneAmp PCR System 9700 thermal cycler (Applied Biosystems, Foster City, CA, USA) according to the manufacturer’s technical manual. To determine optimal PCR conditions for PCR, 1 ng of positive-control 9948 DNA was amplified in triplicate at different cycle numbers (26, 28 and 30) and different annealing temperatures (58 °C, 60 °C and 62 °C).

PCR amplified products were subsequently prepared for CE by adding 1 μL of amplification product to 9 μL of a 19:1 mixture of deionized Hi-DiTM Formamide (Thermo Fisher Scientific, Waltham, MA, USA) and T-500 size standards, then the mixture was denatured at 95 °C for 3 min and subjected to 3 min of cooling on ice. InDel genotyping was performed with capillary electrophoresis on ABI 3500 Genetic Analyzer (Thermo Fisher Scientific) using 36 cm capillary arrays with the POP-4^®^ Polymer (Applied Biosystems, Foster City, CA, USA). Spectral calibration was performed using the J6 Dyeset Template with the 6 Dye Matrix Standards. The electrophoresis conditions involved the following parameters: 24 injection at 1.2 kV, electrophoresis at 15 kV for 1500 s and a run temperature of 60°C. Amplification positive controls (9947A, 9948, 2800M and 007) and negative controls (deionized water) were used to test the overall performance of the multiplex system. Genotyping data were analyzed by GeneMapper^®^ ID-X software (Applied Biosystems) and the analytical threshold was determined by the average and standard deviations of the noise peaks calculated from the 24 negative samples [40].

### Validation studies

#### Repeatability/reproducibility and accuracy testing

Four typical types of positive control DNA (9947A, 9948, 2800M and 007) and 50 unrelated individual samples were amplified in triplicate in two independent accredited laboratories for the repeatability, reproducibility, and concordance study. The genotyping results of the aforementioned samples were compared with those from previously conducted system in our laboratory [38]. Furthermore, pyrosequencing was performed for the accuracy testing of 35InDel kit loci. Single-plex PCR amplification was performed by PyroMark PCR kit (Qiagen) after the biotinylated InDel primers were purified by high performance liquid chromatography (HPLC). The sequencing primers of InDel locus for pyrosequencing were designed by PyroMark Assay Design Software 2.0. Afterwards, the pyrosequencing was performed using PyroMarkTM Q48 instrument (Qiagen) according to the manufacturer’s instructions. PyroMarkTM Q48 Autoprep Software was used for the analysis of pyrosequencing results.

#### Sensitivity study

A sensitivity study is crucial to evaluate the ability to generate reliable genotype profiles of the multiplex kit from a range of target DNA quantities. Serial dilutions of control DNA 9948A were amplified in triplicate with input quantities of 2 ng, 1 ng, 500 pg, 250 pg, 125 pg, 62.5 pg, 31.25 pg and 15.125 pg.

#### Case-type and degraded sample testing

Eleven routine forensic samples including peripheral blood, bloodstain, menstrual blood, umbilical cord blood, saliva, buccal swabs, semen, sperm stain, hair root, nail and muscle were collected for case-type sample testing to validate the practicability of the 35InDel kit. At the same time, to verify the advantage of this kit in the case of degraded samples, DNA from five 13-year-old bloodstains was analyzed by the 35InDel kit and the Huaxia Platinum System (Applied Biosystems) for comparison. The DNA extracted from degraded samples were verified by agarose gel electrophoresis. PCR amplification of Huaxia Platinum System was performed according to manufacturer′s instructions.

#### Stability study

To verify the tolerance of the 35InDel kit against PCR inhibitors, the capacity to obtain genetic data from compromised samples was evaluated by amplifying 1 ng of control DNA 9948 containing different concentrations of five common forensic inhibitors (humic acid, indigotin, hematin, urea and melanin (Aladdin Co. Ltd, Shanghai, China)). The concentration series were as follows: 100 ng/μL, 200 ng/μL, 300 ng/μL, 500 ng/μL, 750 ng/μL and 1 000 ng/μL of humic acid, 5 000 ng/μL, 10 000 ng/μL, 20 000 ng/μL, 30 000 ng/μL, 40 000 ng/μL and 50 000 ng/μL of indigotin, 100 μmol/L, 200 μmol/L, 300 μmol/L, 500 μmol/L, 750 μmol/L, and 1 000 μmol/L, of hematin, 4 000 ng/μL, 8 000 ng/μL, 16 000 ng/μL, 32 000 ng/μL, 64 000 ng/μL and 100 000 ng/μL of urea and 100 ng/μL, 200 ng/μL, 300 ng/μL, 500 ng/μL, 750 ng/μL and 1 000 ng/μL of melanin. Each test was amplified in triplicate.

#### Species specificity

In the case of forensic genetics, DNA of non-human species is encountered occasionally. The 35InDel kit primers are designed to be human-specific with minimal cross-reaction with non-primate animals. Assessing species specificity encompassed testing performance of 35InDel kit in amplifying 5 ng of template DNA from 10 common non-primates (pig, cow, horse, donkey, sheep, dog, mouse, rabbit, chicken and duck) and primates (chimpanzee). Animal blood samples were collected with the approval of the Animal Use Committee of the Academy of Forensic Science, Ministry of Justice, China (SJY2020-W024). Each sample was tested in triplicate.

#### Intra-locus balance

The intra-locus balance of the 35InDel kit was analyzed by genotyping data from 70 South Han Chinese samples. The intra-locus balance of each heterozygote locus is represented by the ratio of low peak height to high peak height. Mean, median, minimum, and maximum of the intra-locus balance were also calculated.

#### Mixture study

To assess the capability of 35InDel kit in DNA mixture deconvolution, control DNA 9948 and 9947A were mixed in various ratios (1:1, 1:9, 1:19) with 1 ng of total DNA template and each of them was amplified in triplicate.

#### Population genetics and statistical analysis

To evaluate the forensic efficiency and polymorphisms of the 35InDel kit, a total of 262 unrelated healthy Southern Han individuals (73 males and 189 females) were investigated. Human blood samples were collected upon approval of the Ethics Committee at the Academy of Forensic Science, Ministry of Justice, China (SJY2020-W024). Written informed consent was acquired from each participant involved in this study. Genomic DNA samples were extracted from peripheral blood with a QIAamp DNA Blood Mini kit (Qiagen). DNA was quantified using a NanoDrop 2000 spectrophotometer and analyzed by NanoDrop 2.4.7c software (NanoDrop Technologies Inc., USA) according to the manufacturer’s recommendations.

Allele frequencies of the 34 autosomal InDels were calculated by counting method. Hardy–Weinberg equilibrium (HWE), Allele frequency distributions and corresponding forensic statistical parameters including homozygosity (Hom), heterozygosity (Het), matching probability (MP), power of discrimination (PD), power of exclusion (PE) and typical paternity index (TPI) of 34 autosomal InDel loci were computed with PowerStats V12.xls based on allelic frequencies. The polymorphism information content (PIC) was also calculated to evaluate the informativeness of the 34 autosomal InDel loci. Power of exclusion in duos (PE_D_) and trios (PE_T_) as well as the combined power of exclusion in duos (CPE_D_) and trios (CPE_T_) were calculated according to the following formula:

$$PE_{D}=\sum_{i=1}^{n}p_{i}\times {\left(1-p_{i}\right)}^{2}+\sum_{i=1}^{n-1}\sum_{j=i+1}^{n}2\times p_{i}\times p_{j}\times {\left(1-p_{i}-p_{j}\right)}^{2}$$

$$PE_{T}=\sum_{i=1}^{n}p_{i}\times {\left(1-p_{i}\right)}^{2}-\frac{1}{2}\times \sum_{i=1}^{n-1}\sum_{j=i+1}^{n}p_{i}^{2}\times p_{j}^{2}\times (4-3\times p_{i}-3\times p_{j})$$

*n* is the number of alleles, *p_i_* is the frequency of allele i on the locus, and *p_j_* is the frequency of allele j on the locus.

$$\mathrm{CPE}=1-\prod_{j=1}^{k}\left(1-{PE}_{j}\right)$$

*k* is the number of InDel markers, and PE_j_ is the PE value of the No. j genetic marker in the 35InDel kit.

Linkage disequilibrium (LD) for all pair wise InDel loci was estimated using the HaploView v4.2 genetics software [41]. *F_st_* values for pairwise interpopulation comparisons were calculated based on allele frequencies of 34 InDels utilizing ARLEQUIN version 3.5 software [42]. Dimensionality reduction statistical analyses (Principal component analysis, PCA) based on allele frequencies were performed in SPSS 20.0 (IBM, Armonk, NY, USA). Test size *α* = 0.05.

## Results and discussion

### Construction and optimization of the 35InDel kit

In this study, 35 InDel loci with alleles length va­ried in 3–30 bp were selected to construct a robust system that can amplify 35 loci simultaneously in one reaction. Detailed information of each locus is shown in Supplementary Table S1. The concentrations of the InDel primers in the final primer mix were adjusted according to the height of the genotyping profiles in the electropherograms. Detailed characteristics of the primers, the concentration of each primer pair, dye labels and amplicon size are shown in Supplementary Table S2.

Size precision is crucial for the accuracy and reliability of genotyping. The evaluation methods were running 24 full injections of the allelic ladder on a 3500 Genetic Analyzer and calculating the standard deviation for each allele. As shown in Supplementary Table S3, the deviations for most loci are almost within 0.05 bases, which ensures accurate allele detection within the bin range.

As shown in Supplementary Figure S1A, for all tested samples, complete profiles were obtained at cycle numbers 26, 28 and 30. The overall balance is not affected by changes in the cycle number while the peak heights increase with the increases of the cycle number. The results suggested that the number of PCR cycles could be changed to adapt to the reaction conditions of different DNA concentrations. For comprehensive consideration, 28 was determined as the optimal cycle number. As shown in Supplementary Figure S1B, complete and accurate profiles could be obtained at annealing temperatures of 58 °C, 60 °C and 62 °C with the average peak height 1 219.49 RFU (58 °C), 7 528.22 RFU (60 °C) and 11 198.61 RFU (62 °C), respectively. The lower or higher average peak height will affect the interpretation of the profiles. Thus, in the present study, the appropriate annealing temperature was maintained at 60 °C for the optimal amplification efficiency. After optimization of the PCR conditions, thermal cycling parameters were determined: initial incubation at 95 °C for 2 min, 28 cycles at 94 °C for 5 s and 60 °C for 2 min, and final extension at 60 °C for 5 min.

**Figure 2.**
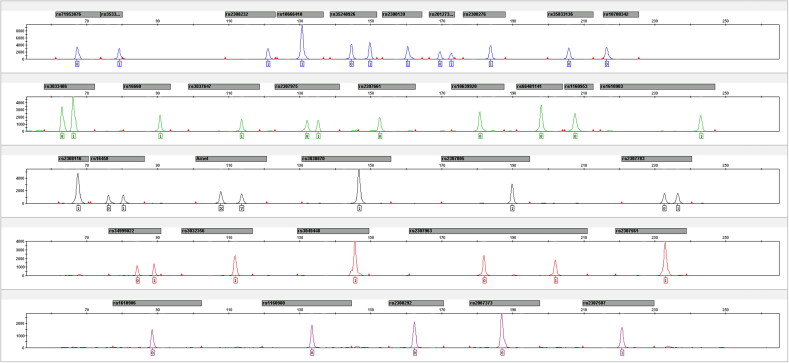
The electropherogram of the 35InDel kit from 1 ng of 9948 sample.

After marker selection, primer design, ladder preparation and optimization of PCR conditions, a novel 35-plex InDels multiplex typing system was constructed. Thirty-four autosomal InDel loci and Amelogenin gender marker could be amplified simultaneously and all loci obtained relatively balanced signal intensities. The analytical threshold was determined as 145 RFU by calculating. The genotyping results of positive control DNA 9947A, 9948, 2800M and 007 are listed in Supplementary Table S1, and the genotyping profiles of DNA 9948 are shown in Figure 2.

### Validation studies

#### Repeatability/reproducibility, concordance and accuracy testing

The acquisition of complete and accordant genotyping profiles of four positive control DNA and 50 individual samples from two independent accredited laboratories has demonstrated excellent reproduci­bility and concordance of 35InDel kit. Moreover, the repeatability of 35InDel kit has also been veri­fied by triplicate tests with the aforementioned samples.

**Figure 3.**
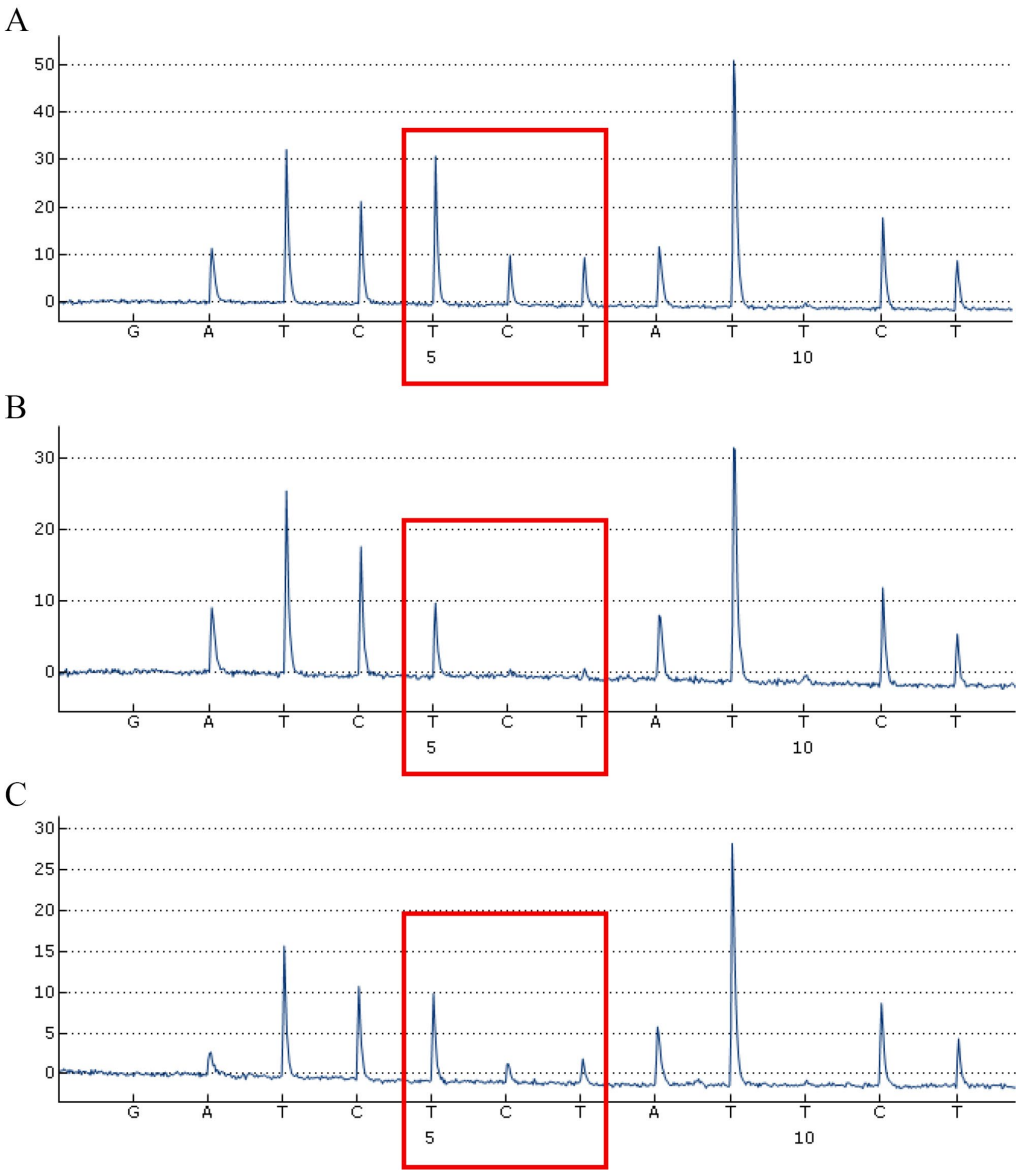
Genotyping of rs2307661 InDel by pyrosequencing. (A) Homozygous genotype with insertions from a South Han Chinese sample. (B) Homozygous genotype with deletions from 9948 DNA. (C) Heterozygous genotype from 2800M DNA. The expected sequence with insertion is ATTTCCT(TTCT)ATTTTTTCCT.

The genotype shown in the 35InDel kit was consistent with the genotype in our previously conducted panel, with an exception for the rs2307661 marker, which displayed a homozygote for the deletion (0,0) in the 9948 sample and a heterozygote (0,1) in the 2800M sample [38]. The accuracy of the rs2307661 InDel genotyping results from 35InDel kit was verified by pyrosequencing. As shown in Figure 3, homozygous samples for insertion show correspondent peaks with the expected insertion sequence and reveal no variation in single nucleotide peak heights (Figure 3A). In homozygote samples for the deletion, the peaks for the InDel sequence are absent (Figure 3B). In heterozygote samples, the peak heights are reduced to half of the signal (Figure 3C). The sequence results of the InDel markers were concordant with the reference FASTA sequence in the NCBI dbSNP database.

#### Sensitivity study

**Figure 4.**
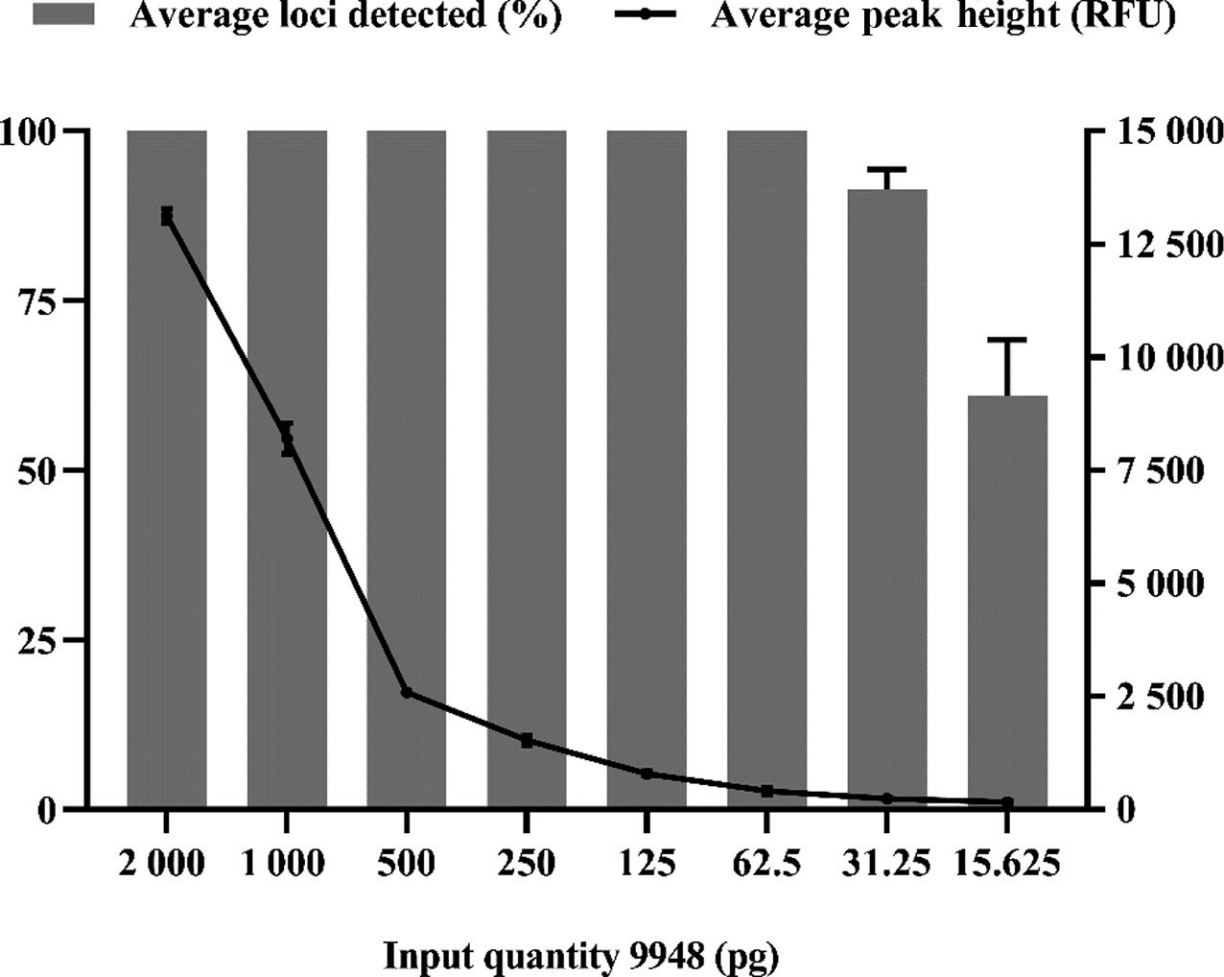
Sensitivity study of the control DNA 9948 positive control sample ranging from 15.625 pg to 2 ng. Histogram and plots of average percentage of loci detected and average peak height of the 35 InDel loci against DNA input quantity. The left Y-axis represents the average percentage (%) of loci detected, and the right Y-axis represents the average peak height (RFU). Error bars represent the standard deviations in triplicate tests.

To determine the upper and lower limits of the 35InDel kit, serial dilutions of the positive control DNA 9948 were amplified in triplicates. As shown in Figure 4, complete genotyping profiles could be observed with the DNA input ranging from 2 ng down to 62.5 pg, with average detected peak heights ranging from 13 120 RFU to 408 RFU, respectively. When the quantity of input DNA was down to 31.25 pg and 15.625 pg, the average loci detection rates declined to 91.43% for 31.25 pg and 65.71% for 15.625 pg, with the average peak heights ranging from 243 RFU to 153 RFU, respectively. These results indicated the optimal DNA input quantity for PCR amplification is above 62.5 pg for the 35InDel kit. This range of input DNA quantities is enough for routine forensic analysis.

#### Case-type sample testing

For the case-type samples testing, 11 kinds of routine forensic samples (peripheral blood, bloodstain, buccal swab, saliva, hair, nail, menstrual blood, umbilical cord blood, semen, sperm stain and muscle) were all genotyped with ideal profiles using the 35InDel kit.

Representative electropherogram obtained from five 13-year-old bloodstain DNA is shown in Supplementary Figure S2A for the 35InDel kit and in Supplementary Figure S2B for the Huaxia Platinum System. For the Huaxia Platinum System, alleles within 300 bp were well-typed. However, alleles with larger fragments showed significantly allele dropout. In contrast, a full profile with relatively balanced peak height was obtained with the 35InDel kit, verifying the stability of the InDel for degraded DNA samples. The results confirmed that the small-amplicon strategy diminishes allele and locus dropout and enhances the ability of complete genotyping from challenging samples with degraded DNA.

#### Stability study

In reality, DNA samples from forensic contexts are always collected with inferior quality due to the different substrates of deposition or complex environmental impact. Humic acid, the major organic constituent of soil, is the major soil inhibitor of DNA profiling [43]. The humic acid may inhibits DNA amplification by targeting the enzyme Taq polymerase required for PCR amplification [44]. Bloodstained denims are common forensic evidentiary material, while the indigo dye co-extracted with DNA from denims may inhibit the PCR reaction [45,46]. In addition, biological samples such as blood, urine and hair contain various PCR inhibitors including hematin, urea and melanin that may be co-extracted with the DNA [47–49]. These PCR inhibitors can negatively affect the DNA typing results by binding to the polymerase or interacting with DNA, which results in partial or complete inhibition of PCR [50–54].

Nevertheless, the makers in 35InDel kit are not susceptible to the effects of inhibitors with studied concentration. Complete profiles could be obtained when humic acid ≤ 1 000 ng/μL, indigotin ≤ 50 000 ng/μL, hematin ≤ 1 000 μmol/L, urea ≤ 100 000 ng/μL or melanin ≤ 1 000 ng/μL. Thus, we have verified that the 35InDel kit is effective in generating profiles from forensic samples with a certain degree of inhibitor influence.

It should be noted that 100–1 000 ng/μL of mela­nin only affected the detection of the fragments with a fixed size (approximately 90–110 bp) and presents abnormal peak shape (Supplementary Figure S3). Similar phenomenon was observed in another test in which 1 μL of different concentrations of melanin was amplified by 35InDel kit without DNA input, while normal profiles could be obtained by adding the same concentration of melanin to the PCR product of positive control DNA 9948. It has been demonstrated that melanin can bind to metal ions and a variety of small molecules, which may explain the specific electrophoretic mobility on 90–110 bp size fragments [53].

#### Species specificity

Among the non-primate species, no PCR amplification was observed for the 10 species over a thre­shold of 145 RFU except for a few abnormal-shaped “OL” peaks with low height (145–299 RFU). Nevertheless, these “OL” peaks detected from non-primate DNAs electrophoretically migrated outside of the allelic ladder which did not interfere with the genotyping results. This testing demonstrated that the kit has very low cross-reactivity with non-primate DNA samples.

Primate DNA from chimpanzee produced partial InDel profiles with several of the peaks detected as off-ladder alleles (Supplementary Figure S4). Twelve of the 35 InDel loci were amplified for chimpanzee sample and all of them were monomorphic alleles. This is not beyond expectation, as a high degree of homology between human and chimpanzee genomes has been reported and the monomorphic alleles in chimpanzees possibly represent the ancestral states of these sequences [55].

#### Intra-locus balance

**Figure 5.**
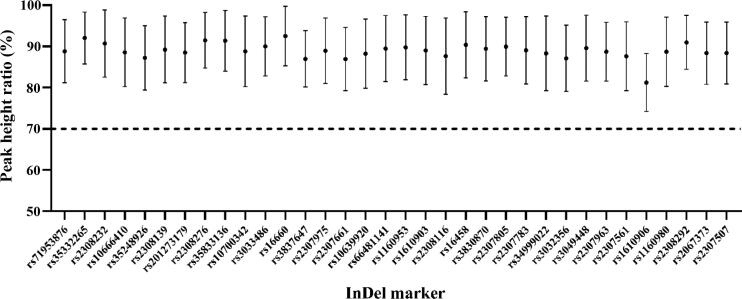
Intra-locus balance of the 35InDel kit. The Y-axis represents the peak height ratio of the heterozygous locus. Error bars represent the standard deviations.

Intra-locus balance of the 35InDel kit was calculated using the detected peak height from typing results of 70 South Han Chinese samples (Supplementary Table S4). As shown in Figure 5, the peak height ratios of the alleles in each heterozygous locus of autosomal InDels are all above 70%, which ensures relatively accurate heterozygote genotyping.

#### Mixture study

DNA mixtures sample are very common in forensic casework. A total of 1 ng template DNA mixed with 9948 and 9947 DNA at various ratios (1:1, 1:9, 1:19,) was tested. The detection of minor alleles was demonstrated by two loci (rs71953876 and rs3032356) in which 9948 and 9947A have non-overlapping alleles (Supplementary Figure S5). At ratios 1:1 and 1:9, two non-overlapping alleles from the minor contributor could be detected. While at ratio of 1:19, the rs3032356 allele from minor contributor were not observed. The results showed that the alleles from minor components could be observed by biallelic genetic markers only in the case of non-overlapping alleles. Multiplex biomarkers such as microhaplotype and DIP-STR may be more efficient in DNA mixture deconvolution.

#### Population investigation and genetic polymorphisms

We further explored the forensic efficiency of the 35InDel kit for population study by genotyping the makers in 262 unrelated healthy Southern Han individuals (73 males and 189 females). Full and clear profiles were obtained, and the allele frequencies and forensic parameters are listed in Supplementary Table S5. All mar­kers were in HWE at a *P* value of more than 0.05 except rs34535242 (*P* = 0.0425). However, after Bonferroni correction was applied (i.e. 0.05/34 =0.001 470 6), all loci were in equilibrium. In addition, as shown in Supplementary Figure S6, LD analysis was performed in the studied populations, and no evidence for linkage disequilibrium was found among the 34 autosomal InDel with the *r^2^*< 0.01. Therefore, the 35InDel kit could be utilized for personal identification and kinship testing.

**Figure 6.**
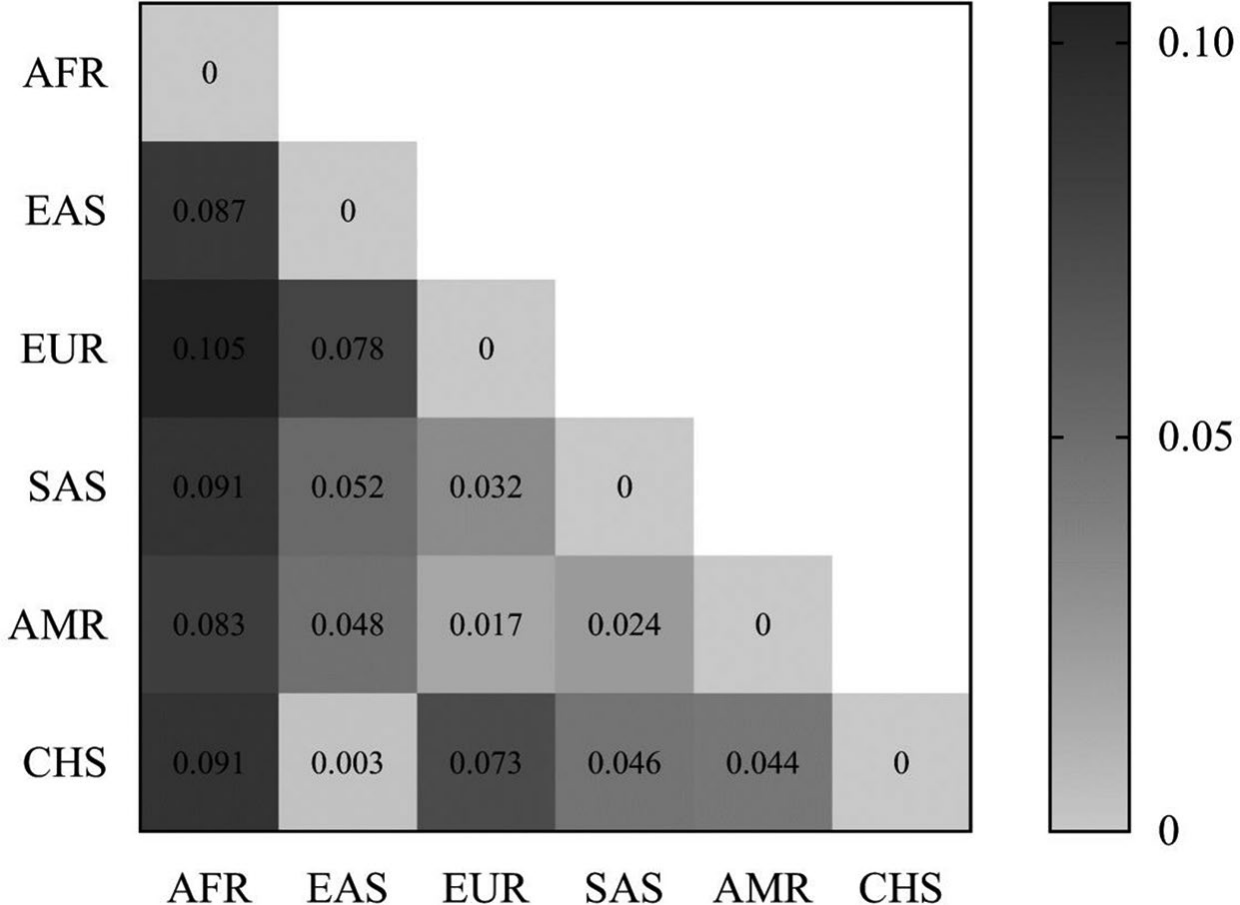
*F_st_* values between studied populations. AFR: African; EAS: East Asian; EUR: European; SAS: South Asian; AMR: American; CHS: South Han Chinese.

For the 34 autosomal InDels, the Het ranged from 0.3855 (rs2308232) to 0.5382 (rs10666410 and rs201273179) with an average of 0.4582. The PIC were all over 0.3 with the variation from 0.3023 (rs2308232) to 0.3749 (rs2307561). The PD ranged from 0.5337 (rs2308232) to 0.6484 (rs2307561). The CPD was 0.999 999 999 999 978 and the CPE_D_ and CPE_T_ were 0.978837 and 0.999573, respectively. Studies previously reported the population study of 30 InDels in the Han population from different areas determined using the Investigator^®^ DIPplex kit [29,31,32,34,56,57]. As shown in Table 1, the CPD was range from 0.999 999 999 964 6 to 0.999 999 999 987 8, which were all lower than the calculated parameters of 35InDel kit. The results showed that our 35InDel kit is more compatible for individual identification in Chinese population than the Investigator^®^ DIPplex kit. Besides, CPD increased as the number of InDel markers included in the multiplex system changed. The CPD of 35InDel kit is higher than that of SifaInDel 45plex system [38], but lower than that of AGCU InDel 50 kit [58]. A relatively low CPE indicates that the 35InDel kit could only be used as a complementary tool for STRs panel in kinship analysis.

**Figure 7.**
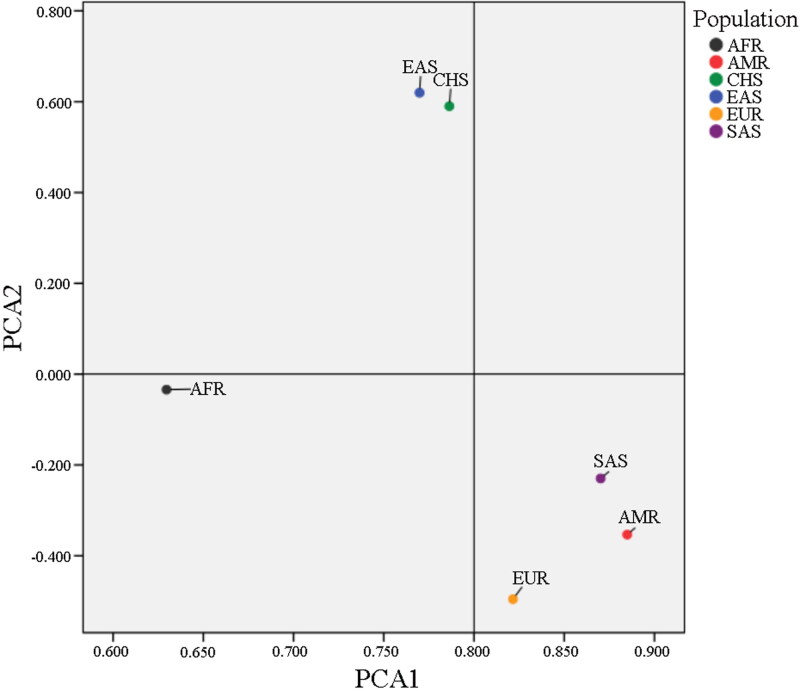
A principal component analysis (PCA) plot showing the genetic relationships between South Han Chinese and other five reference populations. AFR: African; EAS: East Asian; EUR: European; SAS: South Asian; AMR: American; CHS: South Han Chinese.

Moreover, to demonstrate the polymorphism among different populations with 35InDel kit, frequency data of the 34 InDels from African, American, European, East Asian, South Asian were collected from 1000 Genomes Project database (Supplementary Table S6). The pairwise *F_st_* genetic distance values of the five populations are shown in Figure 6. The minimum *F_st_* was observed in pairwise comparison between South Han Chinese and East Asian (*F_st_* = 0.003), which was consistent with the subordination of the South Han Chinese population to the East Asian population at the biogeographic level. Subsequently, to further examine the diversity of the genetic structures among the six populations, a classical nonparametric linear dimensionality reduction technique, PCA was adopted. PCA removes centralized redundancy dimensions of the raw data to maximize the entropy of every dimension. As illustrated in Figure 7, PCA plots indicate a close genetic relationship between South Han Chinese and East Asian, and the results of clustering show that the separation of different populations is clear and definite. These statistical results substantiate the polymorphism and informativeness of the studied InDel.

## Conclusion

In this study, a novel six-dye multiplex InDel kit, the Goldeneye^TM^ DNA ID System 35InDel kit is established and commercialized. Compared to the Investigator^®^ DIPplex kit, the 35InDel kit contains 34 high heterozygous autosomal InDel loci in South Han Chinese and increases the CPD from 0.999 999 999 987 8 to 0.999 999 999 999 978.

A series of developmental validation through a PCR-CE workflow was conducted, confirming that the 35InDel kit is capable of obtaining accurate and stable genotyping with good repeatability, sensitivity and species specificity. Furthermore, polymorphism and independence of the studied InDels in Chinese Han population were verified by the allelic frequencies and statistical parameters obtained from the population investigation. In conclusion, the present results confirmed the superior performance of the Goldeneye^TM^ DNA ID System 35InDel kit in forensic human identification, routine forensic casework as well as degraded samples.

## Supplementary Material

Supplemental Material
