# Supplementary material for: Developmental validation of the novel six-dye GoldeneyeTM DNA ID System 35InDel kit for forensic application
Source: Forensic Sci Res. 2021 Aug 28;7(4):673–84. doi: 10.1080/20961790.2021.1945723 (PMC9930762; doi:10.1080/20961790.2021.1945723)
Supplement: Supplemental Material [file TFSR_A_1945723_SM4316.zip › Supplementary Figure.docx]

# Developmental validation of the novel six-dye Goldeneye^TM^ DNA ID System 35InDel Kit for forensic application

**Supplementary Figure**

**
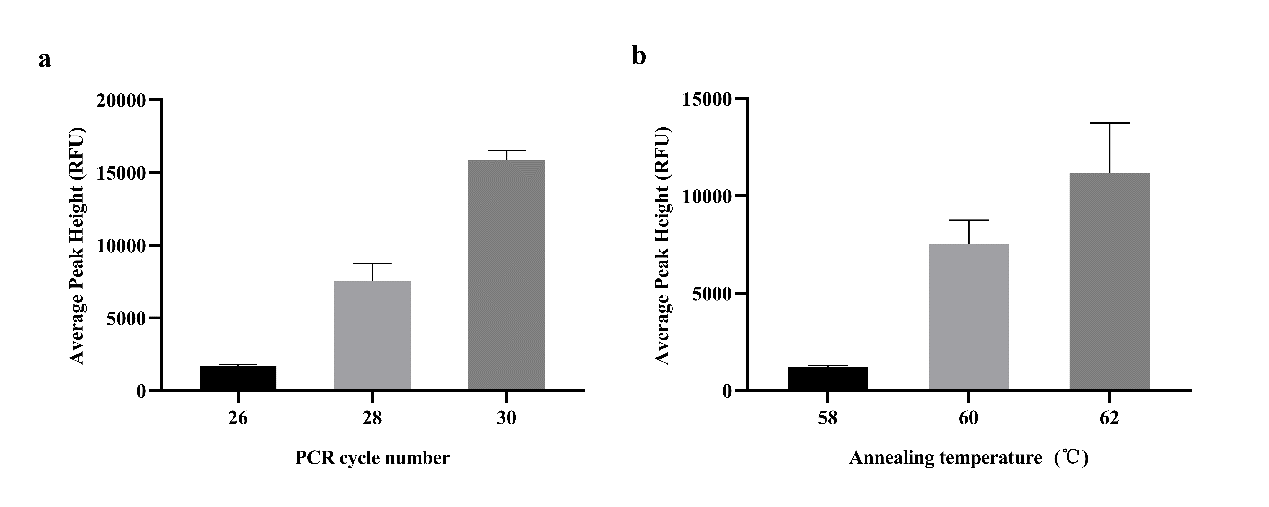
**

**Figure S1.** Average peak height (RFU) at different PCR conditions. (a) Different cycles of 26, 28 and 30 of the 35InDel Kit by amplifying 1 ng of control DNA of 9948 with 60℃ annealing temperature. (b) Annealing temperature at 58℃, 60℃ and 62℃ of the 35InDel Kit by amplifying 1 ng of control DNA of 9948 under 30 cycles. The Y-axis represents the average peak height (RFU). Error bars represents the standard deviations in triplicate tests.


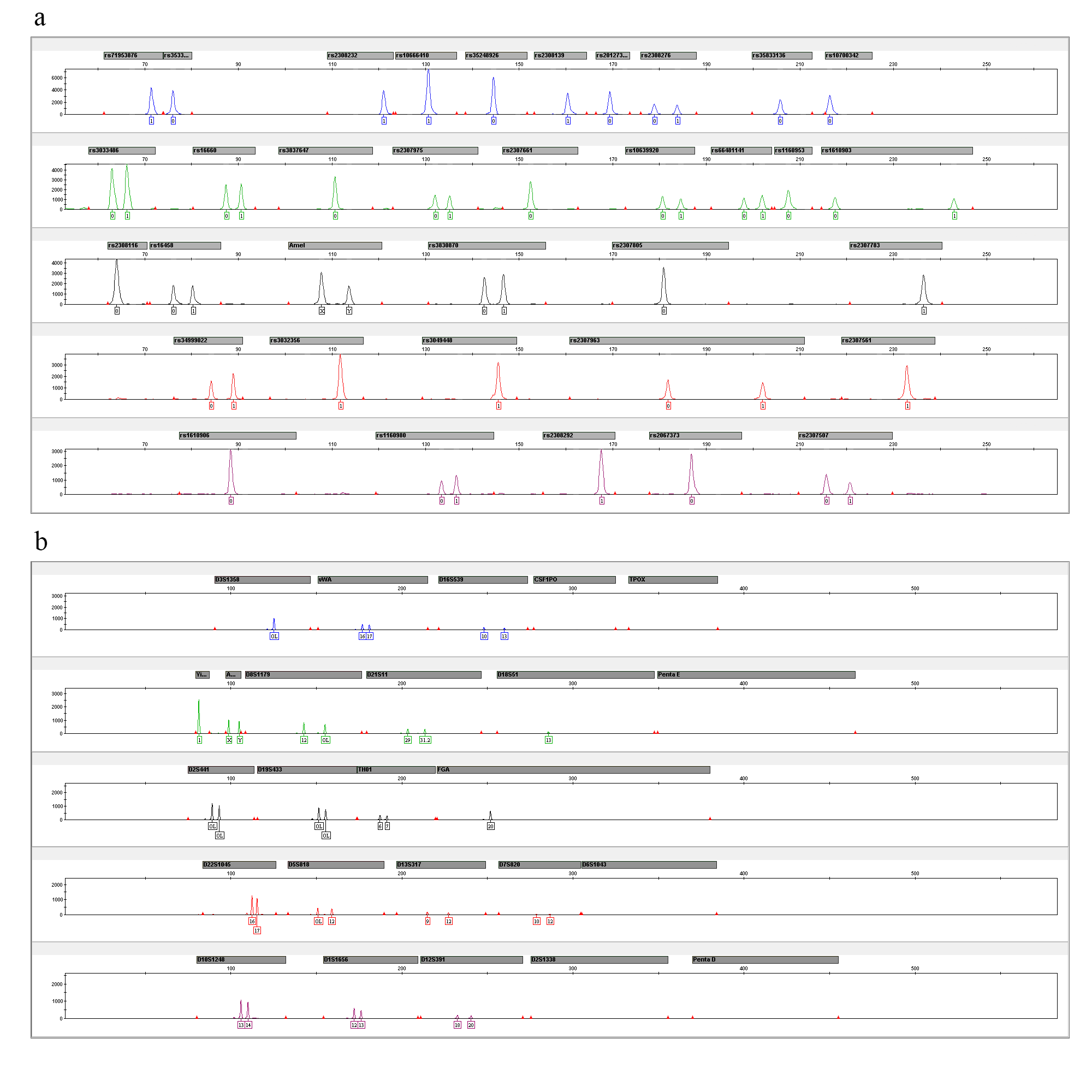


**Figure S2.** Electropherogram of a thirteen-year-old bloodstain sample using (a) the 35InDel Kit and (b) the Huaxia Platinum System.


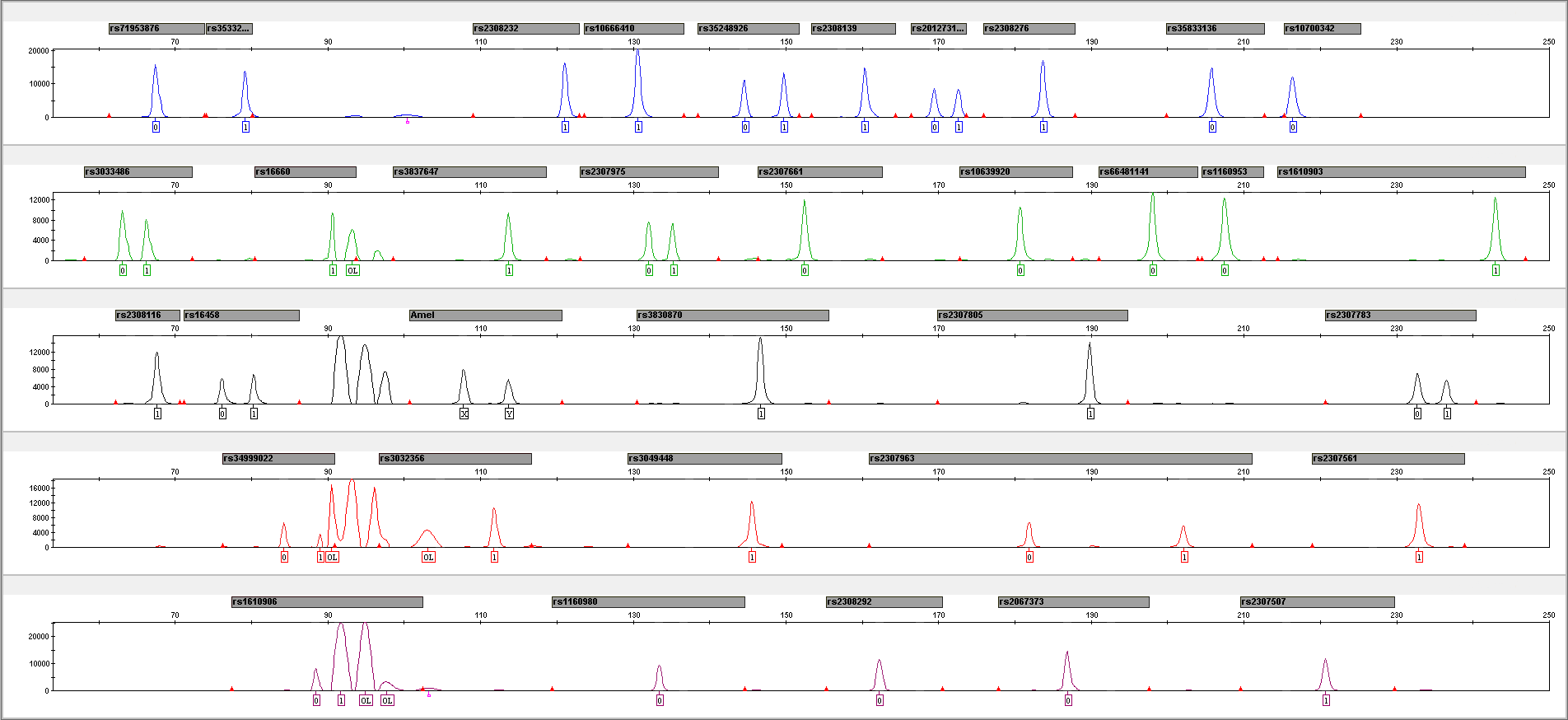


**Figure S3.** Electropherogram of 1000ng/μL of melanin from 9948 DNA.


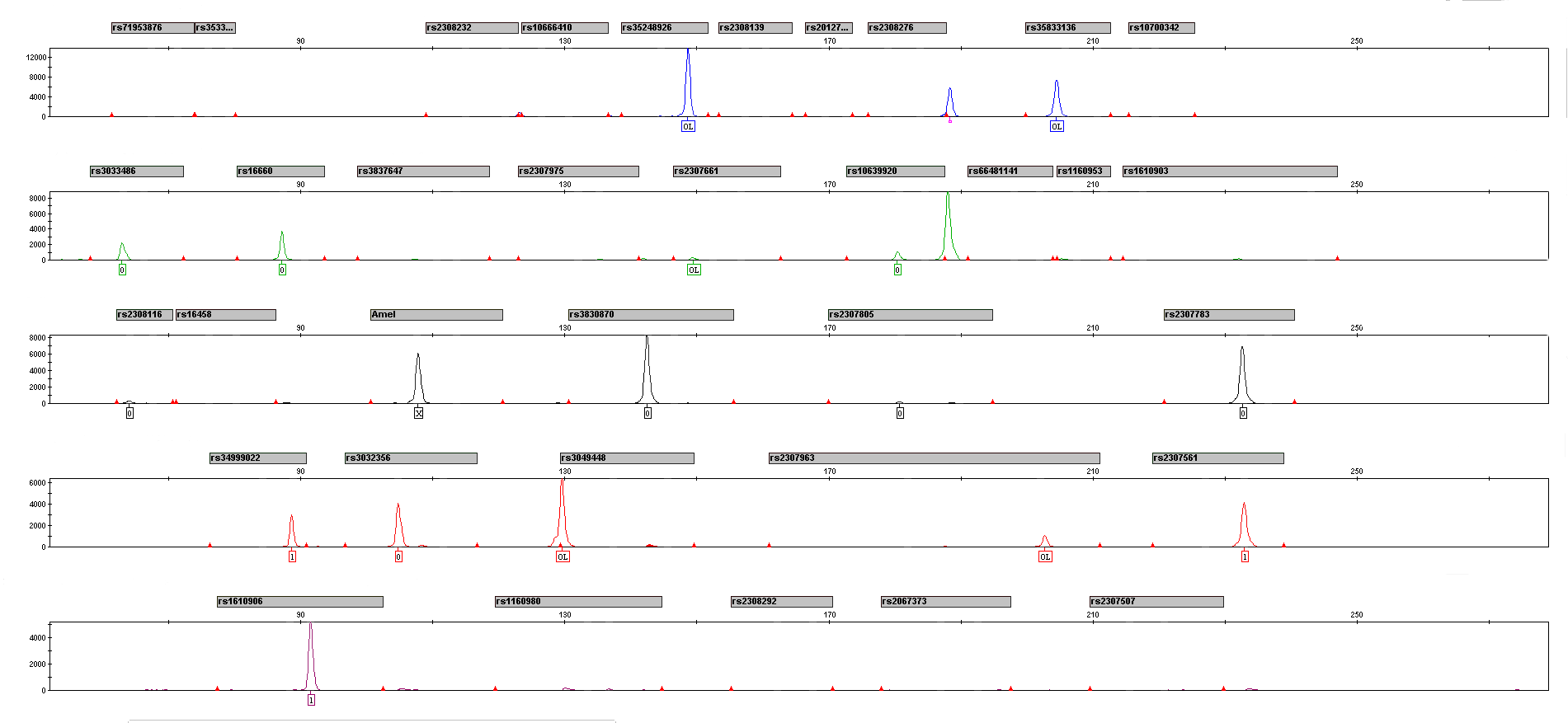


**Figure S4.** Electropherogram of 5 ng of DNA from chimpanzee DNA.


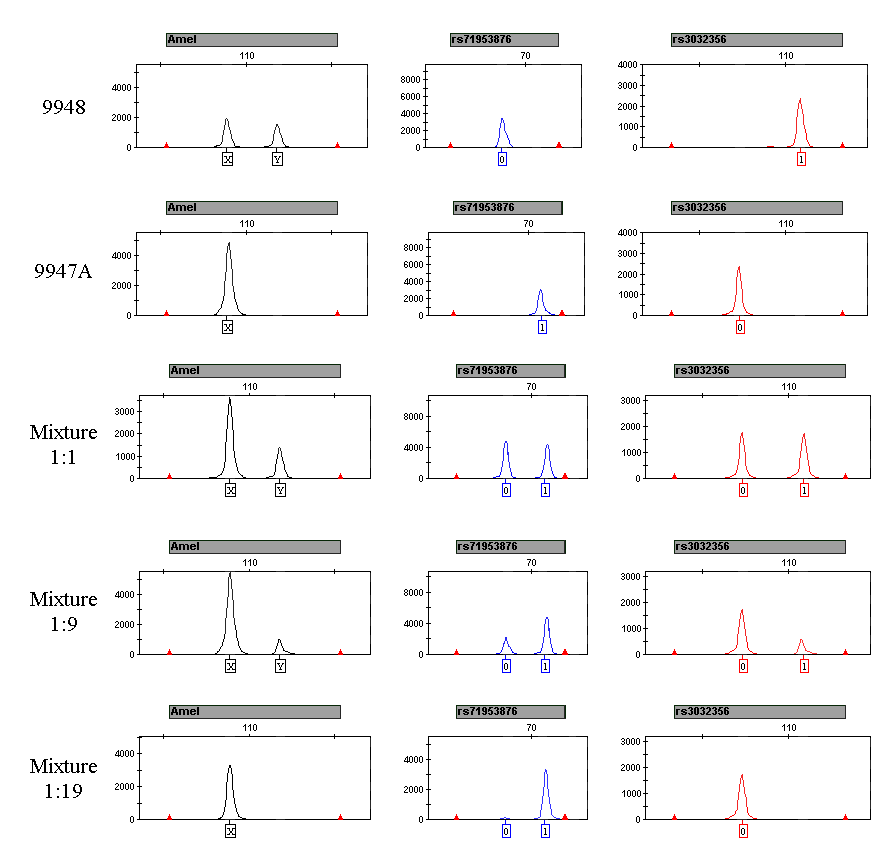


**Figure S5.** Electropherograms of 35InDel Kit in mixture study.


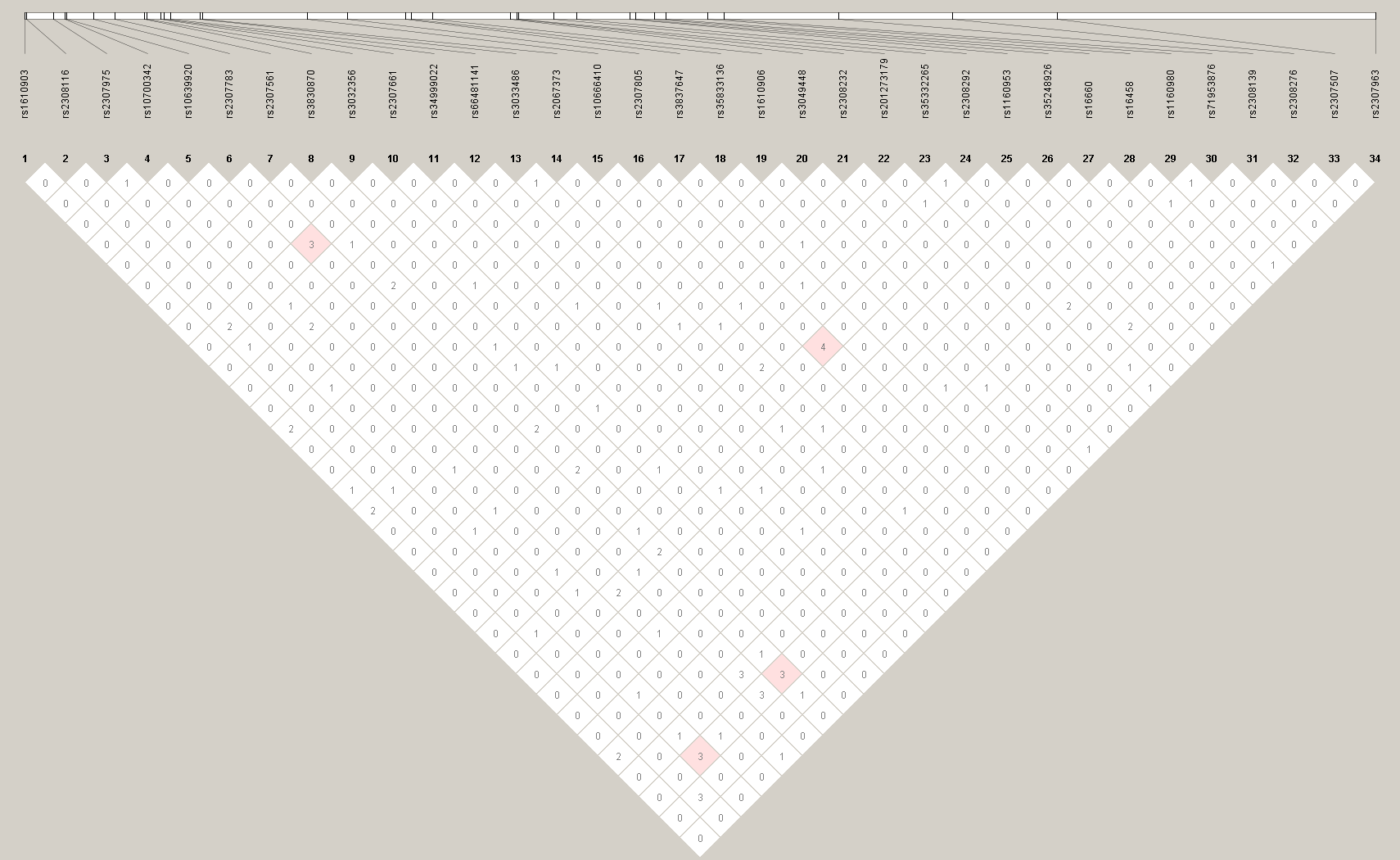


**Figure S6.** Linkage disequilibrium analysis for 34 autosomal InDels in South Han Chinese population.
