# Supplementary material for: Developmental validation of the novel six-dye GoldeneyeTM DNA ID System 35InDel kit for forensic application
Source: Forensic Sci Res. 2021 Aug 28;7(4):673–84. doi: 10.1080/20961790.2021.1945723 (PMC9930762; doi:10.1080/20961790.2021.1945723)
Supplement: Supplemental Material [file TFSR_A_1945723_SM4316.zip › Supplementary Table.docx]

# Developmental validation of the novel six-dye Goldeneye^TM^ DNA ID System 35InDel Kit for forensic application

**Supplementary table**

**Table S1.** Detailed information of the 35 InDel markers and genotyping data of four positive control DNAs.

| **InDel maker** | **Chromosome** | **Physical position**  **(****GRCh38.p12)** | **Allele** | **Genotypes of control DNA** | | | |  |
| --- | --- | --- | --- | --- | --- | --- | --- | --- |
|  |  |  |  | **9947A** | **9948** | **2800M** | **007** | |
| rs71953876 | Chr9 | 132504795-132504798 | -/CACC | 1/1 | 0/0 | 0/1 | 0/1 | |
| rs35332265 | Chr5 | 103097071-103097073 | -/TAC | 1/1 | 1/1 | 1/1 | 1/1 | |
| rs2308232 | Chr12 | 96598108-96598109 | -/AACTTA | 1/1 | 1/1 | 0/0 | 1/1 | |
| rs10666410 | Chr8 | 60278129-60278130 | -/AGTG | 1/1 | 1/1 | 0/0 | 0/0 | |
| rs35248926 | Chr11 | 117181354-117181358 | -/TCTCT | 0/0 | 0/1 | 0/1 | 0/1 | |
| rs2308139 | Chr6 | 152457200-152457202 | -/TAT | 0/1 | 1/1 | 1/1 | 0/1 | |
| rs201273179 | Chr5 | 96993305-96993307 | -/TAT | 0/1 | 0/1 | 0/1 | 0/0 | |
| rs2308276 | Chr2 | 172051073-172051077 | -/TTTAA | 1/1 | 1/1 | 1/1 | 1/1 | |
| rs35833136 | Chr11 | 78286634-78286635 | -/AAATA | 0/0 | 0/0 | 0/0 | 0/0 | |
| rs10700342 | Chr21 | 18333862-18333864 | -/TCA | 0/1 | 0/0 | 0/0 | 1/1 | |
| rs3033486 | Chr21 | 41762791-41762793 | -/AAT | 0/1 | 0/1 | 1/1 | 1/1 | |
| rs16660 | Chr12 | 120463132-120463134 | -/GGT | 1/1 | 1/1 | 0/1 | 0/0 | |
| rs3837647 | Chr14 | 77296603-77296605 | -/AAG | 1/1 | 1/1 | 0/0 | 0/0 | |
| rs2307975 | Chr3 | 16386203-16386205 | -/AAG | 1/1 | 0/1 | 0/1 | 0/1 | |
| rs2307661 | Chr5 | 34893806-34893807 | -/TTCT | 0/1 | 0/0 | 0/1 | 0/0 | |
| rs10639920 | Chr8 | 18571898-18571901 | -/GTAG | 0/1 | 0/0 | 0/1 | 0/0 | |
| rs66481141 | Chr20 | 36687229-36687232 | -/CTTA | 0/0 | 0/0 | 0/0 | 0/0 | |
| rs1160953 | Chr5 | 116290382-116290385 | -/GATA | 0/1 | 0/0 | 0/0 | 0/0 | |
| rs1610903 | Chr5 | 11312686-11312711 | -/ACTCCCCATATACCCTCACCCTCACC | 1/1 | 1/1 | 1/1 | 1/1 | |
| rs2308116 | Chr6 | 11715199-11715201 | -/TCC | 0/1 | 1/1 | 1/1 | 0/1 | |
| rs16458 | Chr7 | 122511277-122511280 | -/CTTC | 0/0 | 0/1 | 0/1 | 0/1 | |
| Amelogenin | Chr X Chr Y | 11296874-11296985  6869841-6869958 | NA | 0/0 | 0/1 | 0/1 | 0/1 | |
| rs3830870 | Chr11 | 32100319-32100322 | -/GCTT | 1/1 | 1/1 | 1/1 | 1/1 | |
| rs2307805 | Chr12 | 67311232-67311240 | -/GTTTATGGG | 1/1 | 1/1 | 0/1 | 1/1 | |
| rs2307783 | Chr7 | 23268017-23268020 | -/GGGT | 1/1 | 0/1 | 1/1 | 1/1 | |
| rs34999022 | Chr18 | 35470354-35470358 | -/TAAAA | 0/1 | 0/1 | 0/1 | 0/0 | |
| rs3032356 | Chr14 | 32474381-32474387 | -/TTCCATC | 0/0 | 1/1 | 0/1 | 0/1 | |
| rs3049448 | Chr13 | 95475154-95475161 | -/GGATCAGA | 0/1 | 1/1 | 0/1 | 1/1 |  |
| rs2307963 | Chr1 | 245388659-245388680 | -/ACCTTAAGGGGGAAAGGGCTAA | 1/1 | 0/1 | 1/1 | 0/1 |  |
| rs2307561 | Chr18 | 26923543-26923548 | -/AGTGTT | 1/1 | 1/1 | 0/1 | 0/1 |  |
| rs1610906 | Chr7 | 82096636-82096638 | -/TAT | 0/1 | 0/0 | 0/0 | 0/1 |  |
| rs1160980 | Chr5 | 129701719-129701721 | -/GAT | 0/1 | 0/0 | 0/0 | 0/0 |  |
| rs2308292 | Chr4 | 106968616-106968620 | -/TAAGT | 0/1 | 0/0 | 0/1 | 1/1 |  |
| rs2067373 | Chr13 | 42124310-42124311 | -/TATATT | 0/1 | 0/0 | 1/1 | 1/1 |  |
| rs2307507 | Chr1 | 190287887-190287891 | -/TTTAT | 0/1 | 1/1 | 0/1 | 0/1 |  |

**Table S2.** Detailed primer information of the 35InDel Kit.

| **InDel marker** | **Primer sequence (5′→3′)** | **Fluorochrome** | **Primer concentration ( µM)** | **Amplicon size (bp)** |
| --- | --- | --- | --- | --- |
| rs71953876 | TTTTACTGAGATGCCCAACCTGC | 6-FAM | 0.15 | 83 |
|  | CAATGATTTTGTCACCGTTTCCT |  |  |  |
| rs35332265 | CTAAGATAGGTTAAGTAATTTGCCGAAGT | 6-FAM | 0.17 | 92 |
|  | GGATTTTGGAATCAAGTAGAGCTGG |  |  |  |
| rs2308232 | TGTCCTTTGGGTTAATCTAAATGGT | 6-FAM | 0.18 | 126 |
|  | TTACTTCCTATTCTCCTTGCTTCGT |  |  |  |
| rs10666410 | TGTGCCTAGTCCTGCTCTGAGATAA | 6-FAM | 0.20 | 138 |
|  | GGCCCAGATGTTGGTCCATAGTCTC |  |  |  |
| rs35248926 | CTTCTGGCGGACCACAAACA | 6-FAM | 0.15 | 160 |
|  | AAGGAGGGCAGGGCTCATAG |  |  |  |
| rs2308139 | TTTGATTAGCTTTAACTTCCTCACTTT | 6-FAM | 0.30 | 170 |
|  | ATCATTGTGGCTTCACTACAGATAATA |  |  |  |
| rs201273179 | TAGAAAGAGGTTCTGCCCAAGTTGTT | 6-FAM | 0.16 | 181 |
|  | ATAAGCAAATTCCCACTTATACAGCA |  |  |  |
| rs2308276 | TGCATGGAATTTCTCCATTTGAGAC | 6-FAM | 0.17 | 189 |
|  | CTTTGTCCAAGAAGTTGCCTGAGTA |  |  |  |
| rs35833136 | TGTCTTTCTCCCCTTTAAGTTCTGAG | 6-FAM | 0.19 | 216 |
|  | TTCTGTAATCCTCTTGCTGTCCTTCT |  |  |  |
| rs10700342 | ACAACAGAGCAGAGGTCTTCCAGC | 6-FAM | 0.15 | 227 |
|  | CTGCATTCTTCAAATGAGATTACTTTTC |  |  |  |
| rs3033486 | CCTAGGCTGGATTCGGCATCTC | HEX | 0.22 | 73 |
|  | CCACACAAAAGGAACTGTAGATTTATTACT |  |  |  |
| rs16660 | AAATCTCATAGTAAGGCAGGACAGG | HEX | 0.12 | 99 |
|  | GAGGTGGTGACTATCCCTTTGGTAC |  |  |  |
| rs3837647 | AGCAAAAGACGAAAAACAACTTGTCC | HEX | 0.18 | 123 |
|  | TGGTTTGTCAAAATGCTGTA |  |  |  |
| rs2307975 | TTTCTTGCCTCTTCACTTTTGCTAG | HEX | 0.18 | 146 |
|  | AGTCTGTTAGTTCTGTGGCTGTCAA |  |  |  |
| rs2307661 | AAGTGTCTCCTCGTGTCTTTCGTAAC | HEX | 0.17 | 162 |
|  | TATTGTATTATTTAGGGATCACGCAG |  |  |  |
| rs10639920 | ACAAAGACATCAAAGTAGCTTCCAAAATC | HEX | 0.26 | 190 |
|  | GTATTTACATAAGCCTCCTTCTGTGGTCA |  |  |  |
| rs66481141 | AAGACAGTGAGGAGTAGATGTTCCAGAC | HEX | 0.17 | 212 |
|  | GCTTCCCTTTGATTTTATTACCGTCTAT |  |  |  |
| rs1160953 | CATGAAGCTAAGTGTACAGGATCAAAT | HEX | 0.28 | 219 |
|  | GTATAGATGATGCTGATTAAATCTTTGG |  |  |  |
| rs1610903 | CCCATACACCACCATCCCACAT | HEX | 0.08 | 230 |
|  | GGAAGGGTAAAATGCCACAAGC |  |  |  |
| rs2308116 | CAGAACTACCAGGGCTGTAAGTTCTG | TAMRA | 1.78 | 74 |
|  | TTGTTGCCAAATACTGTCAATGCTAC |  |  |  |
| rs16458 | CTACCAATTAACAGTCTCAAAGTTTTACA | TAMRA | 0.28 | 84 |
|  | GGCAAATATGTATACTTTGATTATTACTCAT |  |  |  |
| Amelogenin | CCCTGGGCTCTGTAAAGAATAGTG | TAMRA | 0.14 | ChrX:112 ChrY:118 |
|  | CCAACCATCAGAGCTTAAACTGG |  |  |  |
| rs3830870 | TGACTTCTGGCTCATCGCCTTAC | TAMRA | 0.32 | 152 |
|  | GCACCTCTGGGGTTACAGGTACA |  |  |  |
| rs2307805 | AGTAATTGAGCCATACTACCCGAACT | TAMRA | 0.12 | 184 |
|  | AATCCATGTAAAACCTGATAACCCA |  |  |  |
| rs2307783 | TGGTGGTCAATGCCTTTGTTCAG | TAMRA | 0.17 | 243 |
|  | GTTTGGCTTTGTTTGATTTCTGTTG |  |  |  |
| rs34999022 | CTAAAAGGCTAAGATTAACTAATGGCTCT | ROX | 0.17 | 90 |
|  | AAAGGAAATGTAGTACAGAAAGGTAACAT |  |  |  |
| rs3032356 | ATGGAGCCACTTCCTTAACCCCTCA | ROX | 0.1 | 110 |
|  | CCATTCAATTGCCTTTGACCCACA |  |  |  |
| rs3049448 | ACAAATATGTAGAGTGCAAGGGAGCA | ROX | 0.18 | 148 |
|  | GGAAACCTATAAGCCAGGAAGTAGGA |  |  |  |
| rs2307963 | TCCTTGGGCTGGTCCAGTGT | ROX | 0.14 | 207 |
|  | ATGCTGGAAAGGTGGTGCCT |  |  |  |
| rs2307561 | AAGCAAAGAATATCATTCCTGCACTGC | ROX | 0.21 | 237 |
|  | GGGCCACAAATTTTATCTATCTCATCC |  |  |  |
| rs1610906 | AATACCTAGATATCAACATGATGG | VIG | 0.33 | 97 |
|  | TGTTTGGATGTTTTGTTATTGCAGG |  |  |  |
| rs1160980 | CTGCATTGAAGTTGATATTTTTC | VIG | 0.49 | 143 |
|  | ATATGCCCTTGATTATGTTGC |  |  |  |
| rs2308292 | TGTATGTGCACGCATGTCCT | VIG | 0.17 | 172 |
|  | TCTGTTAGGCGCACTGTGTC |  |  |  |
| rs2067373 | ACACTCTCAGCCTCAATAGATTT | VIG | 0.22 | 191 |
|  | GTACCAGCAATTCACACTGAC |  |  |  |
| rs2307507 | TGGCTAGTTATGCACACTTCAG | VIG | 0.19 | 226 |
|  | TTGCTAACAAGTGTTCAAAATGTG |  |  |  |

| **InDel marker** | **Fluorochrome** | **Allele 1** | | **Allele 2** | |
| --- | --- | --- | --- | --- | --- |
|  |  | **Fragment size (bp)** | **Standard deviation** | **Fragment size (bp)** | **Standard deviation** |
| rs71953876 | 6-FAM | 67 | 0.03 | 71 | 0.00 |
| rs35332265 | 6-FAM | 76 | 0.02 | 79 | 0.04 |
| rs2308232 | 6-FAM | 115 | 0.04 | 121 | 0.04 |
| rs10666410 | 6-FAM | 127 | 0.03 | 131 | 0.04 |
| rs35248926 | 6-FAM | 145 | 0.05 | 150 | 0.04 |
| rs2308139 | 6-FAM | 157 | 0.05 | 160 | 0.05 |
| rs201273179 | 6-FAM | 169 | 0.05 | 172 | 0.06 |
| rs2308276 | 6-FAM | 179 | 0.03 | 184 | 0.05 |
| rs35833136 | 6-FAM | 206 | 0.06 | 211 | 0.02 |
| rs10700342 | 6-FAM | 216 | 0.05 | 219 | 0.04 |
| rs3033486 | HEX | 63 | 0.05 | 66 | 0.03 |
| rs16660 | HEX | 87 | 0.02 | 91 | 0.02 |
| rs3837647 | HEX | 111 | 0.02 | 114 | 0.03 |
| rs2307975 | HEX | 132 | 0.02 | 135 | 0.05 |
| rs2307661 | HEX | 152 | 0.04 | 157 | 0.05 |
| rs10639920 | HEX | 181 | 0.03 | 185 | 0.03 |
| rs66481141 | HEX | 198 | 0.04 | 202 | 0.04 |
| rs1160953 | HEX | 207 | 0.04 | 212 | 0.04 |
| rs1610903 | HEX | 217 | 0.03 | 243 | 0.07 |
| rs2308116 | TAMRA | 64 | 0.03 | 68 | 0.03 |
| rs16458 | TAMRA | 76 | 0.02 | 80 | 0.04 |
| Amelogenin | TAMRA | 108 | 0.05 | 114 | 0.04 |
| rs3830870 | TAMRA | 143 | 0.04 | 147 | 0.04 |
| rs2307805 | TAMRA | 181 | 0.01 | 190 | 0.03 |
| rs2307783 | TAMRA | 233 | 0.03 | 236 | 0.03 |
| rs34999022 | ROX | 84 | 0.02 | 89 | 0.03 |
| rs3032356 | ROX | 105 | 0.04 | 112 | 0.03 |
| rs3049448 | ROX | 137 | 0.01 | 146 | 0.04 |
| rs2307963 | ROX | 182 | 0.01 | 202 | 0.06 |
| rs2307561 | ROX | 227 | 0.03 | 233 | 0.03 |
| rs1610906 | VIG | 88 | 0.01 | 91 | 0.02 |
| rs1160980 | VIG | 133 | 0.02 | 137 | 0.02 |
| rs2308292 | VIG | 162 | 0.04 | 167 | 0.03 |
| rs2067373 | VIG | 187 | 0.03 | 192 | 0.04 |
| rs2307507 | VIG | 216 | 0.03 | 221 | 0.05 |

**Table S3.** Size precision of the 35InDel Kit (N = 24).

**Table S4.** Intra-locus balance of each autosomal locus in the 35InDel Kit.

| **InDel marker** | **N.** | **Mean** | **Median** | **Minimum** | **Maximum** |
| --- | --- | --- | --- | --- | --- |
| rs71953876 | 29 | 0.888 | 0.901 | 0.701 | 0.995 |
| rs35332265 | 37 | 0.921 | 0.939 | 0.763 | 0.999 |
| rs2308232 | 26 | 0.907 | 0.934 | 0.715 | 0.996 |
| rs10666410 | 41 | 0.886 | 0.917 | 0.714 | 0.999 |
| rs35248926 | 30 | 0.872 | 0.873 | 0.700 | 0.977 |
| rs2308139 | 33 | 0.892 | 0.887 | 0.715 | 0.999 |
| rs201273179 | 34 | 0.885 | 0.896 | 0.734 | 0.998 |
| rs2308276 | 30 | 0.915 | 0.930 | 0.771 | 0.997 |
| rs35833136 | 31 | 0.914 | 0.942 | 0.734 | 0.996 |
| rs10700342 | 39 | 0.888 | 0.906 | 0.706 | 0.998 |
| rs3033486 | 27 | 0.900 | 0.914 | 0.704 | 0.999 |
| rs16660 | 22 | 0.925 | 0.955 | 0.716 | 0.982 |
| rs3837647 | 25 | 0.870 | 0.865 | 0.752 | 0.976 |
| rs2307975 | 30 | 0.889 | 0.893 | 0.705 | 1.000 |
| rs2307661 | 37 | 0.869 | 0.892 | 0.718 | 0.983 |
| rs10639920 | 28 | 0.882 | 0.897 | 0.706 | 0.991 |
| rs66481141 | 31 | 0.895 | 0.919 | 0.728 | 0.994 |
| rs1160953 | 34 | 0.898 | 0.916 | 0.714 | 0.995 |
| rs1610903 | 29 | 0.890 | 0.908 | 0.707 | 0.998 |
| rs2308116 | 22 | 0.876 | 0.866 | 0.710 | 0.999 |
| rs16458 | 41 | 0.904 | 0.928 | 0.738 | 0.998 |
| rs3830870 | 30 | 0.894 | 0.910 | 0.708 | 0.996 |
| rs2307805 | 29 | 0.900 | 0.909 | 0.708 | 0.985 |
| rs2307783 | 29 | 0.891 | 0.920 | 0.718 | 0.994 |
| rs34999022 | 34 | 0.883 | 0.877 | 0.702 | 0.999 |
| rs3032356 | 28 | 0.871 | 0.887 | 0.701 | 0.986 |
| rs3049448 | 22 | 0.896 | 0.922 | 0.728 | 0.998 |
| rs2307963 | 26 | 0.887 | 0.884 | 0.748 | 0.999 |
| rs2307561 | 37 | 0.876 | 0.894 | 0.702 | 0.997 |
| rs1610906 | 26 | 0.812 | 0.808 | 0.722 | 0.985 |
| rs1160980 | 25 | 0.887 | 0.919 | 0.737 | 0.997 |
| rs2308292 | 36 | 0.910 | 0.924 | 0.715 | 0.991 |
| rs2067373 | 39 | 0.884 | 0.898 | 0.706 | 0.999 |
| rs2307507 | 35 | 0.884 | 0.900 | 0.711 | 0.999 |

N: numbers of observed heterozygotes that were used for intra-locus balance calculations.

**Table S5.** Allelic frequencies and forensic parameters of South Han Chinese population (N = 262).

| **No.** | | **InDel marker** | | **F_I_** | | **Hom** | |  | | **Het** | | **MP** | | **PD** | | **PE_D_** | | **PE_T_** | | **PIC** | | **TPI** | | **HWE-*p*** | |  |
| --- | --- | --- | --- | --- | --- | --- | --- | --- | --- | --- | --- | --- | --- | --- | --- | --- | --- | --- | --- | --- | --- | --- | --- | --- | --- | --- |
| 1 | rs71953876 | | 0.6527 | | 0.5573 | |  | | 0.4427 | | 0.3979 | | 0.6021 | | 0.1153 | | 0.2113 | | 0.3506 | |  | | 0.8973 | | 0.7085 | |
| 2 | rs35332265 | | 0.5840 | | 0.5573 | |  | | 0.4427 | | 0.3654 | | 0.6346 | | 0.0861 | | 0.1860 | | 0.3678 | |  | | 0.8973 | | 0.1534 | |
| 3 | rs2308232 | | 0.7538 | | 0.6145 | |  | | 0.3855 | | 0.4663 | | 0.5337 | | 0.1101 | | 0.2071 | | 0.3023 | |  | | 0.8137 | | 0.6480 | |
| 4 | rs10666410 | | 0.4828 | | 0.4618 | |  | | 0.5382 | | 0.3969 | | 0.6031 | | 0.0689 | | 0.1684 | | 0.3747 | |  | | 1.0826 | | 0.2210 | |
| 5 | rs35248926 | | 0.5363 | | 0.4695 | |  | | 0.5305 | | 0.3943 | | 0.6057 | | 0.1184 | | 0.2137 | | 0.3737 | |  | | 1.0650 | | 0.2970 | |
| 6 | rs2308139 | | 0.6240 | | 0.5229 | |  | | 0.4771 | | 0.3951 | | 0.6049 | | 0.1235 | | 0.2177 | | 0.3591 | |  | | 0.9562 | | 0.8210 | |
| 7 | rs201273179 | | 0.4179 | | 0.4618 | |  | | 0.5382 | | 0.4097 | | 0.5903 | | 0.0887 | | 0.1884 | | 0.3682 | |  | | 1.0826 | | 0.1006 | |
| 8 | rs2308276 | | 0.6603 | | 0.5954 | |  | | 0.4046 | | 0.3923 | | 0.6077 | | 0.1126 | | 0.2092 | | 0.3480 | |  | | 0.8397 | | 0.1442 | |
| 9 | rs35833136 | | 0.3817 | | 0.5725 | |  | | 0.4275 | | 0.3746 | | 0.6254 | | 0.1247 | | 0.2185 | | 0.3606 | |  | | 0.8733 | | 0.1409 | |
| 10 | rs10700342 | | 0.6756 | | 0.5191 | |  | | 0.4809 | | 0.4277 | | 0.5723 | | 0.1174 | | 0.2129 | | 0.3423 | |  | | 0.9632 | | 0.1735 | |
| 11 | rs3033486 | | 0.4122 | | 0.5725 | |  | | 0.4275 | | 0.3620 | | 0.6380 | | 0.1195 | | 0.2146 | | 0.3672 | |  | | 0.8733 | | 0.0602 | |
| 12 | rs16660 | | 0.7347 | | 0.6069 | |  | | 0.3931 | | 0.4489 | | 0.5511 | | 0.1174 | | 0.2129 | | 0.3138 | |  | | 0.8239 | | 0.9317 | |
| 13 | rs3837647 | | 0.4847 | | 0.5191 | |  | | 0.4809 | | 0.3665 | | 0.6335 | | 0.1028 | | 0.2010 | | 0.3748 | |  | | 0.9632 | | 0.5263 | |
| 14 | rs2307975 | | 0.6985 | | 0.5802 | |  | | 0.4198 | | 0.4233 | | 0.5767 | | 0.1150 | | 0.2110 | | 0.3325 | |  | | 0.8618 | | 0.9432 | |
| 15 | rs2307661 | | 0.5382 | | 0.5267 | |  | | 0.4733 | | 0.3656 | | 0.6344 | | 0.1249 | | 0.2187 | | 0.3735 | |  | | 0.9493 | | 0.4229 | |
| 16 | rs10639920 | | 0.5592 | | 0.5687 | |  | | 0.4313 | | 0.3547 | | 0.6453 | | 0.1184 | | 0.2137 | | 0.3715 | |  | | 0.8792 | | 0.0425 | |
| 17 | rs66481141 | | 0.3454 | | 0.5534 | |  | | 0.4466 | | 0.4004 | | 0.5996 | | 0.0961 | | 0.1952 | | 0.3500 | |  | | 0.9034 | | 0.8324 | |
| 18 | rs1160953 | | 0.5725 | | 0.5496 | |  | | 0.4504 | | 0.3644 | | 0.6356 | | 0.0760 | | 0.1759 | | 0.3697 | |  | | 0.9097 | | 0.1949 | |
| 19 | rs1610903 | | 0.6011 | | 0.5458 | |  | | 0.4542 | | 0.3757 | | 0.6243 | | 0.0787 | | 0.1787 | | 0.3646 | |  | | 0.9161 | | 0.3949 | |
| 20 | rs2308116 | | 0.2729 | | 0.5916 | |  | | 0.4084 | | 0.4449 | | 0.5551 | | 0.1054 | | 0.2032 | | 0.3181 | |  | | 0.8452 | | 0.7213 | |
| 21 | rs16458 | | 0.4122 | | 0.4733 | |  | | 0.5267 | | 0.4048 | | 0.5952 | | 0.1114 | | 0.2082 | | 0.3672 | |  | | 1.0565 | | 0.1821 | |
| 22 | rs3830870 | | 0.7061 | | 0.5725 | |  | | 0.4275 | | 0.4316 | | 0.5684 | | 0.1215 | | 0.2161 | | 0.3289 | |  | | 0.8733 | | 0.7021 | |
| 23 | rs2307805 | | 0.3645 | | 0.5153 | |  | | 0.4847 | | 0.4044 | | 0.5956 | | 0.1022 | | 0.2005 | | 0.3560 | |  | | 0.9704 | | 0.5045 | |
| 24 | rs2307783 | | 0.7385 | | 0.5611 | |  | | 0.4389 | | 0.4639 | | 0.5361 | | 0.0990 | | 0.1977 | | 0.3116 | |  | | 0.8912 | | 0.0839 | |
| 25 | rs34999022 | | 0.5744 | | 0.5153 | |  | | 0.4847 | | 0.3788 | | 0.6212 | | 0.1237 | | 0.2178 | | 0.3694 | |  | | 0.9704 | | 0.8682 | |
| 26 | rs3032356 | | 0.6660 | | 0.5534 | |  | | 0.4466 | | 0.4077 | | 0.5923 | | 0.1073 | | 0.2048 | | 0.3459 | |  | | 0.9034 | | 0.9780 | |
| 27 | rs3049448 | | 0.6279 | | 0.5611 | |  | | 0.4389 | | 0.3828 | | 0.6172 | | 0.1235 | | 0.2177 | | 0.3581 | |  | | 0.8912 | | 0.3425 | |
| 28 | rs2307963 | | 0.6985 | | 0.5878 | |  | | 0.4122 | | 0.4214 | | 0.5786 | | 0.1198 | | 0.2148 | | 0.3325 | |  | | 0.8506 | | 0.7479 | |
| 29 | rs2307561 | | 0.5115 | | 0.5573 | |  | | 0.4427 | | 0.3516 | | 0.6484 | | 0.1180 | | 0.2134 | | 0.3749 | |  | | 0.8973 | | 0.0607 | |
| 30 | rs1610906 | | 0.4008 | | 0.5496 | |  | | 0.4504 | | 0.3736 | | 0.6264 | | 0.0887 | | 0.1884 | | 0.3650 | |  | | 0.9097 | | 0.3177 | |
| 31 | rs1160980 | | 0.3569 | | 0.5305 | |  | | 0.4695 | | 0.4021 | | 0.5979 | | 0.1006 | | 0.1991 | | 0.3537 | |  | | 0.9424 | | 0.7562 | |
| 32 | rs2308292 | | 0.5382 | | 0.4885 | |  | | 0.5115 | | 0.3838 | | 0.6162 | | 0.1248 | | 0.2186 | | 0.3735 | |  | | 1.0234 | | 0.6641 | |
| 33 | rs2067373 | | 0.4179 | | 0.4847 | |  | | 0.5153 | | 0.3965 | | 0.6035 | | 0.0746 | | 0.1745 | | 0.3682 | |  | | 1.0315 | | 0.3679 | |
| 34 | rs2307507 | | 0.6126 | | 0.5229 | |  | | 0.4771 | | 0.3897 | | 0.6103 | | 0.1092 | | 0.2064 | | 0.3620 | |  | | 0.9562 | | 0.9600 | |

F_I_: Frequence of Insertion Allele; Hom: Homozygosity; Het: Heterozygosity; MP: Matching Probability; PD: power of discrimination; PE_D_: power of exclusion in duos; PE_T_: Power of exclusion in trios; PIC: polymorphism information content; TPI: typical paternity index; HWE-*p*: *p* value of Hardy–Weinberg equilibrium.

**Table S6.** Allele frequencies of the 35 InDels in South Han Chinese population and five populations.

| **InDel marker** | **allele** | **AFR** | **EAS** | **EUR** | **SAS** | **AMR** | **CHS** |
| --- | --- | --- | --- | --- | --- | --- | --- |
|  |  | **(n=1322)** | **(n=1008)** | **(n=1006)** | **(n=978)** | **(n=694)** | **(n=262)** |
| rs71953876 | ins | 0.5310 | 0.6480 | 0.6950 | 0.6810 | 0.6700 | 0.6527 |
| rs35332265 | ins | 0.8729 | 0.5506 | 0.7187 | 0.8660 | 0.7780 | 0.5840 |
| rs2308232 | ins | 0.7560 | 0.6870 | 0.6760 | 0.6810 | 0.7350 | 0.7538 |
| rs10666410 | ins | 0.8555 | 0.4782 | 0.5298 | 0.6300 | 0.5350 | 0.4828 |
| rs35248926 | ins | 0.4340 | 0.5450 | 0.2030 | 0.4740 | 0.3130 | 0.5363 |
| rs2308139 | ins | 0.8370 | 0.6850 | 0.7830 | 0.7160 | 0.8000 | 0.6240 |
| rs201273179 | ins | 0.4050 | 0.4730 | 0.4750 | 0.4920 | 0.4150 | 0.4179 |
| rs2308276 | ins | 0.4000 | 0.6010 | 0.6330 | 0.5580 | 0.5130 | 0.6603 |
| rs35833136 | ins | 0.3018 | 0.4018 | 0.1769 | 0.2900 | 0.3240 | 0.3817 |
| rs10700342 | ins | 0.6891 | 0.6270 | 0.6431 | 0.5520 | 0.5070 | 0.6756 |
| rs3033486 | ins | 0.5567 | 0.3085 | 0.6332 | 0.5710 | 0.5850 | 0.4122 |
| rs16660 | ins | 0.9470 | 0.7390 | 0.6480 | 0.6630 | 0.6040 | 0.7347 |
| rs3837647 | ins | 0.7769 | 0.5129 | 0.6193 | 0.4710 | 0.5300 | 0.4847 |
| rs2307975 | ins | 0.3926 | 0.6815 | 0.6193 | 0.9040 | 0.6670 | 0.6985 |
| rs2307661 | ins | 0.5050 | 0.5090 | 0.5540 | 0.7170 | 0.5760 | 0.5382 |
| rs10639920 | ins | 0.3930 | 0.4890 | 0.3570 | 0.4290 | 0.3780 | 0.5592 |
| rs66481141 | ins | 0.4590 | 0.3310 | 0.2120 | 0.3510 | 0.3100 | 0.3454 |
| rs1160953 | ins | 0.8050 | 0.6140 | 0.3330 | 0.4250 | 0.4600 | 0.5725 |
| rs1610903 | ins | 0.5000 | 0.5833 | 0.8579 | 0.7760 | 0.6630 | 0.6011 |
| rs2308116 | ins | 0.2920 | 0.2798 | 0.8310 | 0.5960 | 0.7200 | 0.2729 |
| rs16458 | ins | 0.5760 | 0.4240 | 0.3970 | 0.3350 | 0.4680 | 0.4122 |
| rs3830870 | ins | 0.9190 | 0.7160 | 0.8450 | 0.8000 | 0.8240 | 0.7061 |
| rs2307805 | ins | 0.3440 | 0.3180 | 0.6470 | 0.5140 | 0.5620 | 0.3645 |
| rs2307783 | ins | 0.2320 | 0.7490 | 0.6210 | 0.6150 | 0.6200 | 0.7385 |
| rs34999022 | ins | 0.4387 | 0.6250 | 0.3509 | 0.2880 | 0.4390 | 0.5744 |
| rs3032356 | ins | 0.5197 | 0.6875 | 0.4453 | 0.6560 | 0.4370 | 0.6660 |
| rs3049448 | ins | 0.4947 | 0.6081 | 0.5119 | 0.4900 | 0.6440 | 0.6279 |
| rs2307963 | ins | 0.9289 | 0.6875 | 0.9016 | 0.7980 | 0.9280 | 0.6985 |
| rs2307561 | ins | 0.7481 | 0.5060 | 0.7296 | 0.7790 | 0.5730 | 0.5115 |
| rs1610906 | ins | 0.3464 | 0.3929 | 0.4374 | 0.3800 | 0.4500 | 0.4008 |
| rs1160980 | ins | 0.6233 | 0.3690 | 0.3817 | 0.4490 | 0.3890 | 0.3569 |
| rs2308292 | ins | 0.5265 | 0.5228 | 0.6600 | 0.7180 | 0.6530 | 0.5382 |
| rs2067373 | ins | 0.8056 | 0.4871 | 0.6451 | 0.5430 | 0.5160 | 0.4179 |
| rs2307507^1^ | ins | 0.7700 | 0.6180 | 0.5150 | 0.6120 | 0.5280 | 0.6126 |

AFR: African; EAS: East Asian; EUR: European; SAS: South Asian; AMR: American; CHS: South Han Chinese. Frequency data of other populations was collected from 1000 Genomes Project phase3.

^1^ Frequency data of rs2307507 was not found in 1000 Genomes, and the data was from the genome aggregation database (gnomAD). The sample size of rs2307507 in African, East Asian, European, South Asian and American was 20955, 1556, 32192, 1521 and 6794, respectively.
